# True *versus* Apparent Malaria Infection Prevalence: The Contribution of a Bayesian Approach

**DOI:** 10.1371/journal.pone.0016705

**Published:** 2011-02-18

**Authors:** Niko Speybroeck, Nicolas Praet, Filip Claes, Nguyen Van Hong, Kathy Torres, Sokny Mao, Peter Van den Eede, Ta Thi Thinh, Dioni Gamboa, Tho Sochantha, Ngo Duc Thang, Marc Coosemans, Philippe Büscher, Umberto D'Alessandro, Dirk Berkvens, Annette Erhart

**Affiliations:** 1 Research Institute Health and Society (IRSS), Université Catholique de Louvain la Neuve, Brussels, Belgium; 2 Institute of Tropical Medicine (ITM), Antwerp, Belgium; 3 National Institute of Malariology, Parasitology and Entomology (NIMPE), Hanoi, Vietnam; 4 Institute of Tropical Medicine Alexander von Humboldt, Universidad Peruana Cayetano Heredia (IMT-AvH-UPCH), Lima, Peru; 5 Centre National de Malariologie (CNM), Phnom Penh, Cambodia; 6 Departamento de Bioquimica, Biologia Molecular y Farmacologia, Faculyad de Ciencias y Filosofia, Universidad Peruana Cayetano Heredia, Lima, Peru; Dirección General de Epidemiología, Peru

## Abstract

**Aims:**

To present a new approach for estimating the “true prevalence” of malaria and apply it to datasets from Peru, Vietnam, and Cambodia.

**Methods:**

Bayesian models were developed for estimating both the malaria prevalence using different diagnostic tests (microscopy, PCR & ELISA), without the need of a gold standard, and the tests' characteristics. Several sources of information, i.e. data, expert opinions and other sources of knowledge can be integrated into the model. This approach resulting in an optimal and harmonized estimate of malaria infection prevalence, with no conflict between the different sources of information, was tested on data from Peru, Vietnam and Cambodia.

**Results:**

Malaria sero-prevalence was relatively low in all sites, with ELISA showing the highest estimates. The sensitivity of microscopy and ELISA were statistically lower in Vietnam than in the other sites. Similarly, the specificities of microscopy, ELISA and PCR were significantly lower in Vietnam than in the other sites. In Vietnam and Peru, microscopy was closer to the “true” estimate than the other 2 tests while as expected ELISA, with its lower specificity, usually overestimated the prevalence.

**Conclusions:**

Bayesian methods are useful for analyzing prevalence results when no gold standard diagnostic test is available. Though some results are expected, e.g. PCR more sensitive than microscopy, a standardized and context-independent quantification of the diagnostic tests' characteristics (sensitivity and specificity) and the underlying malaria prevalence may be useful for comparing different sites. Indeed, the use of a single diagnostic technique could strongly bias the prevalence estimation. This limitation can be circumvented by using a Bayesian framework taking into account the imperfect characteristics of the currently available diagnostic tests. As discussed in the paper, this approach may further support global malaria burden estimation initiatives.

## Introduction

Though malaria remains a major public health problem worldwide, particularly for the poorest countries [1], a decreasing trend of its burden, including in sub-Saharan Africa, has been recently reported [2]. Such a change has been attributed to large-scale indoor residual spraying (IRS) campaigns [3] to IRS together with the distribution of insecticide-treated bed net (ITN) [4], and to the introduction of artemisinin-based combination treatments (ACT) together or not with ITNs [5]. These encouraging results are probably due to the increased attention malaria is receiving and the corresponding mobilization of resources. There has also been a recent and radical shift from control to elimination with eventually eradication as a goal, first proposed by the Melinda and Bill Gates Foundation in 2007 and then rapidly endorsed by the World Health Organisation (WHO) and the Roll Back Malaria (RBM) Partnership. The latter developed a Global Malaria Action Plan (GMAP) for a substantial and sustained reduction of the malaria burden in the near and mid-term, and when new tools would make it possible, the eventual global eradication in the long term (http://www.rollbackmalaria.org/gmap/). Within this context, being able to estimate with confidence the malaria prevalence in a given country/district is essential for targeting control/elimination efforts, monitoring the progress towards established goals, e.g. the Millennium Development Goals, and documenting achievements [6]. Without an accurate estimation, established with one or several diagnostic method, one has difficulties in setting and reaching objectives, ordering diagnostics and interventions, and attracting funding agencies that are often result-focused. Microscopy is often taken as the gold standard for diagnosis. However, considering that its sensitivity is limited by low parasite densities, a common feature in low endemic areas, it is a rather imperfect one. In addition, the technique is laborious and requires experienced laboratory technicians, both for the staining and for the reading of the slides so that often, in field conditions, its sensitivity is even lower than expected [7], [8]. Over the past two decades, alternative diagnostic tests have been developed [9] and their sensitivity and specificity have been evaluated against the less-than-optimal reference microscopy test [10]–[14]. In a different approach, no gold standard was designated [9] and the evaluation was done according to the methods described by Hui and Walter [15] that assumed a single, true but unobserved prevalence for each study and common sensitivity and specificity of each diagnostic test across the group of studies [9]. Such assumption may not be true as the sensitivity and specificity may vary according to external factors, which can be field related, e.g., sampling season, age, presence of other cross-reacting diseases [16], [17], and laboratory related factors, e.g., in case of microscopy the experience of the readers [9].

The other assumption made by Hui and Walter is that diagnostic tests are conditionally independent given the true (but latent) prevalence of infection. From a practical point of view, such assumptions can be incorrect and several approaches to circumvent them have been suggested [18], [19].

The performance of different diagnostic techniques depends on the malaria species, the parasite density, previous treatment, gametocytaemia and the quality of the diagnostic method [20]. Test sensitivity and specificity, as traditionally defined, are thus purely theoretical concepts, not necessarily established in the conditions where the test is actually used. In a given setting, the local characteristics (season, presence of cross-reacting organisms, experience of the laboratory technicians) should be considered when obtaining an “adjusted” estimate of the test's sensitivity and specificity [21]. The purpose of this paper is to present a methodological framework for estimating a “true prevalence” and evaluating optimally, in a single analysis different malaria diagnostic tests.

Given that the diagnostic tests' characteristics, i.e. sensitivity and specificity, can be variable and context-specific and that no gold standard is available, combining all available information can be an interesting approach. Indeed, results obtained by different diagnostic tests are related more or less closely to the ”true” malaria prevalence so that it may be useful to estimate it by considering the results obtained by all tests available. As already shown [19], this is only possible by combining test results with expert opinions on the tests' characteristics. In addition, conflicts can be checked by identifying any difference occurring both among experts and between the expert opinions and the actual results, leading to the optimal estimation of the “true” malaria prevalence and of the context-specific diagnostic test characteristics. New diagnostic tests could be easily inserted into this model and their unknown characteristics estimated. With this approach, the prevalence estimations can be optimized (i.e. “true”) and made comparable across different settings, i.e. a site specific analysis would be possible. This is important as there is the need of standardizing malaria prevalence estimates.

## Materials and Methods

### Estimating the malaria prevalence without a reference (gold standard) diagnostic test

The concept of analyzing multiple diagnostic tests can be explained by as an example assuming that only two diagnostic tests are available. The model also assumes that there is a single true but unknown prevalence of *P. falciparum* infection, i.e., a case is defined as a currently infected individual, and that the sensitivity and specificity of the two diagnostic tests are unknown. The class of models in which the infection status is unknown is sometimes referred to as latent class models, i.e. the infection status is latent as it exists but is not evident or detected by a diagnostic test. In the analysis of the diagnostic test characteristics, the following notations can be used (with j = 1, 2…indicating diagnostic test 1, diagnostic test 2,…) and “|”meaning conditional on (or “given”):

- infection status D: D = 1: infected, D = 0: not infected
- test result: Yj = 1: positive, Yj = 0: negative
- sensitivity of test j (Sej): P(Yj = 1 | D = 1)
- specificity of test j (Spj): P(Yj = 0 | D = 0)
- prevalence of infection in the study population (π): P (D = 1)

Assuming independence of Y1 and Y2 and given the infection status, then:
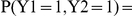


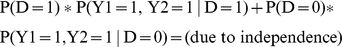


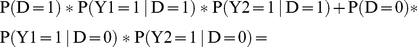


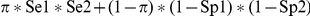



Expanding this to all possible outcomes of two tests' results in a set of 4 equations ( = model):
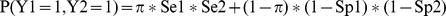


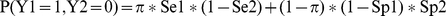


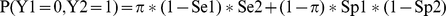


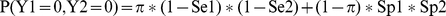



This provides 3 independent equations (because the sum of all left-side proportions in the equations sum to 1) and 5 parameters to estimate. In mathematical terms this is consequently not estimable but it would be when fixing in a deterministic way some parameters or using in a probabilistic way prior information for some of them, e.g., inclusion of the experts' opinions. This approach allows the incorporation of knowledge, such as historical information from experiments similar or related to the one under study, an educated guess about outcomes or even subjective beliefs of the investigator (i.e. expert opinion). These prior probabilities are then updated in a rational way after data collection. Bayesian statistics have recently experienced an explosive growth, with many applications in different areas. Powerful computers and software packages as R and WinBUGS are now common and Bayesian statistics theory is now commonly applied in the development of powerful algorithms and models that process data in new ways [22].

Extending the example to 3 tests will result in 7 independent equations and 7 parameters to estimate, meaning that the equations are “estimable”. With 4 or more tests, there are more equations than parameters and the number of estimable parameters exceeds that to estimate. The model (set of equations) is then over specified. Table 1 shows for 1 to 5 tests, the maximum number of estimable parameters and the number of parameters to be estimated in the absence or presence of conditional independence as a function of the number of tests per subject.

**Table 1:** Maximum number of estimable parameters, number of parameters to be estimated in the absence and presence of conditional independence as a function of the number of tests per subject.

Number of tests	Maximum number of estimable parameters	Parameters to be estimated under conditional dependence	Parameters to be estimated under conditional independence
1	1	3	3
2	3	7	5
3	7	15	7
4	15	31	9
5	31	63	11

For many years, it has been assumed that two (or more) diagnostic tests are conditionally independent given the true (but latent) prevalence of infection [15], [23], i.e. (see before), P(Y1 = 1, Y2 = 1 | D = 1)  =  P(Y1 = 1 | D = 1)*P(Y2 = 1 | D = 1), and that this applies to other possible test results and to disease/infection-free subjects. However, when the two diagnostic tests have a similar biological basis, as is often the case, the conditional independence assumption is untenable [18]. It is possible to insert conditional dependence into the model in several ways [19], but this always entails the need to estimate more parameters then the available equations permit (even with four or more tests). Consequently, the need for a Bayesian approach (i.e. prior information) in these circumstances is even more relevant.

### The Bayesian philosophy and diagnostic testing

Since none of the diagnostic tests included in this study can be considered as the gold standard, a Bayesian approach can be used, i.e. combining data with prior information to estimate the malaria prevalence and the test characteristics. In this paper, a multinomial Bayesian model adapted from Berkvens et al. [19] was used (WinBUGS-code available upon request). This method has been validated for a number of pathogens, e.g. cysticercosis [24] and campylobacter [25]. Prior distributions on the parameters from experts' opinions can be obtained in several ways [26], [27] but the approach used in [19] is well adapted to the way experts think about the test's diagnostic performances as they often know them in relation to a reference test (very often one with a very high specificity). The approach has certain mathematical advantages as well. The final priors of the model are presented in Table 2.

**Table 2:** Prior information (uniform distributions) based on expert opinion.

Parameters	Constrains
Sensitivity of the microscopy for the detection of infected individuals	[0.7–1]
Specificity of the microscopy for the detection of non-infected individuals	[0.9–1]
Probability to have a positive result for the ELISA if the individual is infected and positive for the microscopy	[0.7–1]
Probability to have a positive result for the ELISA if the individual is infected and negative for the microscopy	[0–1]
Probability to have a negative result for the ELISA if the individual is not infected and negative for the microscopy	[0–1]
Probability to have a negative result for the ELISA if the individual is not infected and positive for the microscopy	[0–1]
Probability to have a positive result for the PCR if the individual is infected and positive for the microscopy and the ELISA**	[0.98–1]
Probability to have a positive result for the PCR if the individual is infected, positive for the microscopy and negative for the ELISA**	[0.98–1]
Probability to have a positive result for the PCR if the individual is infected, negative for the microscopy and positive for the ELISA**	[0.75–1]
Probability to have a positive result for the PCR if the individual is infected and negative for the microscopy and the ELISA**	[0.75–1]
Probability to have a negative result for the PCR if the individual is not infected and negative for the microscopy and the ELISA**	[0.95–1]
Probability to have a negative result for the PCR if the individual is not infected, negative for the microscopy and positive for the ELISA**	[0.9–1]
Probability to have a negative result for the PCR if the individual is not infected, positive for the microscopy and negative for the ELISA**	[0.9–1]
Probability to have a negative result for the PCR if the individual is not infected and positive for the microscopy and the ELISA**	[0.9–1]

ELISA = Enzyme-Linked Immunosorbent Assay; PCR = Polymerase Chain Reaction.

**for Vietnam. Peru Iquitos and Peru Jaen only.

Within the multi-diagnostic Bayesian framework, there are mostly more parameters to estimate than equations, especially when conditional dependence is taken into account. This requires the inputs from experts for some of the parameters, i.e. the sensitivity and specificity. They are asked to provide both their estimations and an expression of uncertainty (i.e. credibility intervals). In this study, prior information on the test characteristics was obtained from four experts at the Institute of Tropical Medicine, Antwerp, Belgium, and was expressed as conditional probabilities.

The match between the experts' opinions, any other prior information and the observations can be evaluated through the Bayesian p-value (Bayes-p), the Deviance Information Criterion (DIC) [28] and the number of parameter effectively estimated by the model (pD) [19] which quantifies the impact of the constraints.

The correspondence between the pD and DIC values calculated in the posterior mean of the multinomial probabilities and in the posterior mean of the parameters of the model (parent nodes) was checked. The trend of the Bayes-p [29] towards 0 when narrowing the constraints on the estimates was determined.

The analysis was done in WinBUGS 4 and R 2.11.0. Three chains, 20,000 iterations, following a burn-in of 5,000 were used to assess the convergence of the results. The sensitivity and specificity of the diagnostic tests and the malaria prevalence were estimated by the model. The prevalence was defined as the proportion of individuals infected by *P. falciparum*. The credibility intervals for differences between the characteristics of the diagnostic tests in different conditions with both limits having the same sign (i.e., zero not included in the interval) can be interpreted as the equivalent of a significant difference in a frequentist approach.

### Diagnostic Tests Used

To detect specific antibodies against *P. falciparum* infections, an antibody detection ELISA test was used using a specific antigen for *Plasmodium. falciparum* (GLURP, conserved region R2) (Claes et al. 2010, submitted). For the detection of the specific Plasmodium species DNA, a semi-nested multiplex PCR was used as described by [30]. Samples showing a specific *P. falciparum* amplicon of 395 bp were considered positive while samples showing no amplification or a PCR product of a different size (indicating infection with another species) were considered negative. Samples showing mixed infections of *P. falciparum* with other species were considered positive. A parasitological diagnosis was performed using standard microscopic reading of thick and thin blood films. Details of slide preparation and reading procedures were published elsewhere [31]. Samples were considered negative when no asexual form was found after reading 1000WBC.

### Study areas and ethical clearance

The 3 diagnostic tests were performed on blood samples obtained from individuals living in 3 different countries, Vietnam, Cambodia and Peru, the latter contributing with 2 sites while samples from Cambodia were obtained in the same site but at 2 different time points. Therefore, 5 different datasets were used for this analysis. All the named institutional review boards or ethics committees specifically approved the study. Each study protocol had been reviewed by the ethical committee of the ITM and University of Antwerp as well as by respective national ethical committees for each country (Peru, Cambodia, Vietnam).

### Vietnam

Samples were collected during a cross sectional survey carried out in November-December 2004 (end of the rainy season) in 33 rural communities located in 2 forested districts of Ninh Thuan Province, Central Vietnam. Malaria transmission in the study area was relatively low but perennial with 2 peaks (May-June & October-November), with the sylvatic species *Anopheles. dirus sensu stricto* being the main vector. The survey was part of a community-based trial aimed at determining the effectiveness of long-lasting insecticidal hammocks (LLIH) in preventing forest malaria [31]. Following the trial protocol, over 4,000 individuals (aged 2 to 60 years) were randomly selected from the census for the survey. The trial protocol was approved by the Ethical Committees of the National Institute of Malariology, Parasitology & Entomology, Hanoi, Vietnam, and by the Institutional Review Board of the Institute of Tropical Medicine and the Ethical Committee of the University Hospital, both in Antwerp, Belgium.

### Peru (Jaen)

The cross sectional survey was carried out in April-May 2006 (end of rainy season), in the peri-urban area of Jaen city, the capital of Cajamarca Dept, Northern Peru. Households were chosen randomly and all family members screened for malaria parasites in order to reach a total sample size of 504 individuals (age 6 months–50 years). This study was submitted for ethical approval to the Ethics Review Board of the Universidad Peruana Cayetano Heredia, Lima, Peru (Code SIDISI: 051675).

### Peru (Iquitos)

This study was carried out in several communities (peri-urban & rural) near Iquitos City, the capital of Loreto Department, situated in the Peruvian Amazon, within the activities of the Multi-Country Malaria Project “Malaria control on the cross border areas of the Andean Region: A community based approach” - PAMAFRO together with the National Malaria Control Program. Blood samples were collected in all febrile patients presenting at their health posts or health centers between November and December 2006 (beginning of the rainy season). This study was submitted for ethical approval to the Ethical Review Committee of the Universidad Peruana Cayetano Heredia (EC-UPCH), Lima.

### Cambodia

A malariometric surveys were conducted in August-September (rainy season – survey 1 (S1)), and in November-December 2005 (end of rainy season - survey2 (S2)) in 12 villages located in the forest areas of north-east (Rattanakiri -6 villages) and west Cambodia (Pailin -3 villages; Pursat 3 villages). A representative sample of the inhabitants of each village has been examined (tick film, filter papers, questionnaire, symptoms). The protocol was approved by the Commission of Medical Ethics of the Prince Leopold Institute of Tropical Medicine (Ref: 05 10 4 491).

In all surveys, selected individuals were explained in the local language the study objectives, methodology, risks and benefits, and were asked to give their informed consent. In both Peruvian studies, written informed consent was given by all study participants.

In Vietnam and Cambodia, verbal informed consent was given by all study participants. The institutional review board of the Institute of Tropical Medicine approved this verbal consent.

Positive patients were treated according to the national guidelines. Blood samples for microscopic examination (thick and thin blood film) and for later genotyping and serology on filter paper (Whatman filter paper grade 3) were collected. PCR analysis was not carried out on the Cambodia samples.

## Results

The apparent prevalence was relatively low in all sites, with ELISA giving the highest estimates (Table 3), as confirmed by the higher number of positive individuals for ELISA among all positives by any test (Table 4). According to the Bayes-p estimations, the initial prior information was in agreement with the tests' results for all countries except for Vietnam, where both the constraint on the sensitivity of the microscopy and the experts' opinion on the probability of a positive ELISA in an infected individual with a positive blood slide, did not match the actual results. Both were relaxed from a [0.7–1] to a [0.4–1] uniform distribution (implying less knowledge or more uncertainty than the initial information) to allow agreement between the prior information and the Vietnamese data. After this adaptation all models converged.

**Table 3:** Apparent prevalence figures of *P. falciparum* (exact binomial 95% CI) for the different diagnostic tests by survey.

Sites	Estimated prevalence % (95%CI)		
	Microscopy	ELISA	PCR
**Vietnam**	0.11 (0.10–0.12)	0.24 (0.22–0.26)	0.14 (0.12–0.15)
**Peru (Iquitos)**	0.04 (0.02–0.07)	0.14 (0.10–0.18)	0.03 (0.01–0.05)
**Peru (Jaen)**	0 (0–0.01)	0.11 (0.09–0.14)	0 (0–0.01)
**Cambodia S1**	0.04 (0.03–0.05)	0.11 (0.09–0.12)	-
**Cambodia S2**	0.03 (0.02–0.04)	0.21 (0.19–0.24)	-

ELISA = Enzyme-Linked Immunosorbent Assay; PCR = Polymerase Chain Reaction; S1 = survey 1; S2 = survey 2.

**Table 4:** Number of individuals according to the results of 2 or 3 diagnostic tests by survey.

Diagnostic tests			Number of individuals				
Microscopy	ELISA	PCR	Vietnam	Peru (Iquitos)	Peru (Jaen)	Cambodia S1	Cambodia S2
0	0	0	1530	292	447	1147	752
0	0	1	100	1	0		
0	1	0	351	35	58	129	191
0	1	1	73	1	0		
1	0	0	57	1	0	42	15
1	0	1	59	2	0		
1	1	0	48	5	0	12	15
1	1	1	87	6	0		

0 = negative test result. 1 = positive test result; number = number of individuals for each result category; S1 = survey 1; S2 = survey 2.

Several significant differences were observed between the test characteristics in the 5 different conditions. Notably, the sensitivities of microscopy and ELISA were statistically lower in Vietnam than in Peru-Iquitos, Peru-Jaen and Cambodia (S1 and S2). Similarly, except for the ELISA in Cambodia S2, the specificity estimates for microscopy, ELISA and PCR were significantly lower in Vietnam compared to the corresponding ones in the other sites. The estimated true prevalence was significantly higher in Vietnam than in the other 4 sites among which Peru-Iquitos had the highest prevalence (Table 5).

**Table 5:** Estimated prevalence and diagnostic tests' sensitivity and specificity by survey.

Country	Estimated true prevalence	Estimated sensitivity and specificity					
		Microscopy		ELISA		PCR	
		*se*	*sp*	*se*	*sp*	*se*	*sp*
**Vietnam**	**0.12**	**0.53**	**0.95**	**0.55**	**0.80**	**0.95**	**0.97**
CI lower 95% limit	0.01	0.42	0.94	0.42	0.78	0.89	0.95
CI upper 95% limit	0.15	0.70	0.96	0.70	0.82	1.00	1.00
**Peru Iquitos**	**0.03**	**0.90**	**0.98**	**0.79**	**0.88**	**0.98**	**0.99**
CI lower 95% limit	0.01	0.72	0.96	0.62	0.84	0.95	0.98
CI upper 95% limit	0.05	1.00	0.99	0.95	0.91	1.00	1.00
**Peru Jaen**	**0.00**	**0.89**	**1.00**	**0.85**	**0.88**	**0.98**	**1.00**
CI lower 95% limit	0.00	0.71	1.00	0.64	0.85	0.95	0.99
CI upper 95% limit	0.01	1.00	1.00	1.00	0.91	1.00	1.00
**Cambodia S1**	**0.01**	**0.89**	**0.96**	**0.85**	**0.90**		
CI lower 95% limit	0.00	0.71	0.95	0.63	0.88		
CI upper 95% limit	0.02	1.00	0.98	1.00	0.91		
**Cambodia S2**	**0.01**	**0.89**	**0.98**	**0.84**	**0.79**		
CI lower 95% limit	0.00	0.71	0.96	0.62	0.77		
CI upper 95% limit	0.03	1.00	0.99	1.00	0.82		

CI = credibility interval; Se = sensitivity; Sp = specificity; ELISA = Enzyme-Linked Immunosorbent Assay; PCR = Polymerase Chain Reaction; S1 = survey 1; S2 = survey 2.

A comparison between the “true” and apparent prevalence provides the degree of bias when this is estimated with only one diagnostic test. In Vietnam and Peru, microscopy was closer to the “true” estimate than the other 2 tests (Tables 3 and 5) while ELISA, with its lower specificity, usually overestimated the true infection prevalence (Table 5).

## Discussion

An analysis of three tests for the detection of a malaria infection and for estimating its prevalence was conducted using a Bayesian framework. Bayesian techniques become exceedingly useful for improved interpretation of diagnostic test performance in both the medical and veterinary fields [26], [27]. The Bayesian paradigm clearly corresponds with the way of thinking of most scientists and policy makers alike. Indeed, as results will never been interpreted without any conscious or unconscious reflections, such a process is formalized by a Bayesian framework. This is particularly useful in the context of multiple diagnostic tests because it allows combining different sources of information. A drawback of this approach is the limited number of studies using several diagnostic tests. However, within the Bayesian philosophy even the results of one diagnostic test can be integrated in global estimations as far as the uncertainty is properly acknowledged. It is important to notice that the results (i.e. the true prevalence) depend on opinions obtained from experts who need to be familiar with both malaria and the tests used. In addition, the modelers need to known how to process properly the prior information. Using Bayesian measures of goodness-of-fit, i.e. the DIC and Bayes-p values appropriately guarantee that the different parts of information (i.e., data and expert opinion) are not conflicting, resulting in optimal estimates. The approach corresponds with complex non-linear statistical models where initial values are required. It should be noted that the degree of freedom experts have in expressing opinions will decrease with increasing number of diagnostic tests.

The characteristics of the diagnostic tests employed were estimated without a gold standard. This contrasts with the common practice of estimating the sensitivity and specificity of a test by comparing its results with those obtained by microscopy [13], [14], an inappropriate reference [7], [8]. In addition, both sensitivity and specificity can be influenced by context-specific factors.

The expert opinions originally provided for the 3 tests were not in agreement with the data so that constrains (i.e. the level of “uncertainty”) on the sensitivity of microscopy and ELISA in Vietnam had to be relaxed. This adaptation allowed agreement between prior information and actual data and resulted in a significantly lower sensitivity for the microscopy in Vietnam compared to the other regions, confirming the high variability of this test's sensitivity that depends both on the parasite density and on the experience and skills of the slide reader. Indeed in Vietnam, the parasite density is usually low and PCR data indicate a high proportion of sub-patent infections, as well as mixed infections. Therefore, sensitivity and specificity of a given test can vary according to the setting and such variability can explain the wide confidence intervals reported in other studies [9]. When including in the analysis the variability of the tests' sensitivity and specificity, the malaria prevalence estimations are optimized and become comparable across different settings, i.e. a site-specific analysis can be done.

In Peru-Iquitos, comparable results were obtained with both microscopy and PCR. This finding can be attributed to the sampling of symptomatic patients in whom parasite density is usually higher than in individuals, often healthy, selected randomly for a population survey. The latter infections are asymptomatic, hence with a lower parasite density, or even sub-patent, i.e. undetectable by microscopy.

Combining several diagnostic tests does not imply that the results of each separate test do not have any value. Although positive serological tests may reflect persisting antibodies in non-infected individuals, and hence the lower specificity, they have an advantage of providing an indication of individuals having had a past infection, a valuable information in areas where malaria transmission is very low.

The approach based on the Bayesian framework may be useful to re-examine data obtained with several malaria diagnostic tests [32], [33]. Estimating the malaria prevalence in a specific setting is not straightforward, particularly when considering the lack of a gold standard [9], [34] and the variability of the diagnostic tests' characteristics. A possible solution is combining the results obtained with different tests. Indeed, assessing different diagnostic techniques and estimating their sensitivity and specificity with the assumption that none of them can provide perfect results can be done through the integration of several sources of information. Ochola and colleagues [9] were the first to point out the benefits of combining results of several diagnostic tests for estimating malaria prevalence. Nevertheless, the assumptions inherent to their method are questionable [15], resulting in wide confidence intervals that may in fact reflect real differences in the sensitivity and specificity between different settings [9].

The recent shift from control to elimination with eventually eradication as a goal will require a rigorous assessment of the disease (i.e., infection) free state. Combining information from different diagnostic tests may respond to this need and provide an assessment on the uncertainty related to a disease (i.e., infection) situation. Moreover, the approach based on the Bayesian framework could be used for future studies and the obtained “latent” prevalence could then fit nicely within the global malaria initiatives such as the malaria atlas project (MAP) [35], which is already using the advantages of a Bayesian context. Indeed, currently results are often not comparable from one location to another because different diagnostics tests are used. Without a gold standard, a standardized Bayesian approach for estimating the “true” malaria prevalence can further strengthen the current international efforts towards assessing and reducing the global malaria burden.
